# The Zhu-Lu formula: a machine learning-based intraocular lens power calculation formula for highly myopic eyes

**DOI:** 10.1186/s40662-023-00342-5

**Published:** 2023-06-01

**Authors:** Dongling Guo, Wenwen He, Ling Wei, Yunxiao Song, Jiao Qi, Yunqian Yao, Xu Chen, Jinhai Huang, Yi Lu, Xiangjia Zhu

**Affiliations:** 1 Eye Institute and Department of Ophthalmology, Eye & ENT Hospital, Fudan University, Shanghai, 200031 China; 2 NHC Key Laboratory of Myopia, Fudan University, Shanghai, China; 3 Key Laboratory of Myopia, Chinese Academy of Medical Science, Shanghai, China; 4 Shanghai Key Laboratory of Visual Impairment and Restoration, Shanghai, China; 5 University of Illinois at Urbana-Champaign, Illinois, USA; 6 Shanghai Aier Eye Hospital, Shanghai, China; 7 Eye Hospital of Wenzhou Medical University, Wenzhou, China

**Keywords:** Machine learning, IOL power calculation, High myopia, Prediction error

## Abstract

**Background:**

To develop a novel machine learning-based intraocular lens (IOL) power calculation formula for highly myopic eyes.

**Methods:**

A total of 1828 eyes (from 1828 highly myopic patients) undergoing cataract surgery in our hospital were used as the internal dataset, and 151 eyes from 151 highly myopic patients from two other hospitals were used as external test dataset. The Zhu-Lu formula was developed based on the eXtreme Gradient Boosting and the support vector regression algorithms. Its accuracy was compared in the internal and external test datasets with the Barrett Universal II (BUII), Emmetropia Verifying Optical (EVO) 2.0, Kane, Pearl-DGS and Radial Basis Function (RBF) 3.0 formulas.

**Results:**

In the internal test dataset, the Zhu-Lu, RBF 3.0 and BUII ranked top three from low to high taking into account standard deviations (SDs) of prediction errors (PEs). The Zhu-Lu and RBF 3.0 showed significantly lower median absolute errors (MedAEs) than the other formulas (all *P* < 0.05). In the external test dataset, the Zhu-Lu, Kane and EVO 2.0 ranked top three from low to high considering SDs of PEs. The Zhu-Lu formula showed a comparable MedAE with BUII and EVO 2.0 but significantly lower than Kane, Pearl-DGS and RBF 3.0 (all *P* < 0.05). The Zhu-Lu formula ranked first regarding the percentages of eyes within ± 0.50 D of the PE in both test datasets (internal: 80.61%; external: 72.85%). In the axial length subgroup analysis, the PE of the Zhu-Lu stayed stably close to zero in all subgroups.

**Conclusions:**

The novel IOL power calculation formula for highly myopic eyes demonstrated improved and stable predictive accuracy compared with other artificial intelligence-based formulas.

**Supplementary Information:**

The online version contains supplementary material available at 10.1186/s40662-023-00342-5.

## Background

Modern cataract surgery has shifted to more of a refractive procedure [1, 2]. Currently, intraocular lens (IOL) power calculations have undergone continuous refinement, and improved outcomes have been reached in normal eyes using the newer generation formulas [3, 4]. However, due to the extreme axial length (AL) elongation and complicated ocular biometric characteristics [5], accurate IOL power prediction in highly myopic eyes remains a significant challenge.

Techniques using artificial intelligence (AI) have been applied to improve the accuracy of IOL power calculations. The AI-based formulas, such as Emmetropia Verifying Optical (EVO), Kane, Ladas, Pearl-DGS, Radial Basis Function (RBF) 3.0 and Sramka formulas were recently introduced and have achieved better predictability [2, 6–9]. The Barrett Universal II (BUII) formula has been around for a longer time [10] and have so far showed promising results and is widely used because of its availability on biometers [11].

However, when used on highly myopic eyes, there are still some inaccuracies. On the one hand, these formulas were derived from training datasets of the Caucasian eyes with a whole AL range, which compromised relatively small percentages of highly myopic eyes due to the lower prevalence of high myopia in western countries [12]. Unexpected refractive surprise may still become a problem when applying the existing AI-based formulas to highly or extremely myopic eyes [13, 14]. On the other hand, some of these formulas have maximum AL input limits and target refraction limits. For example, the Kane and RBF 3.0 formulas have maximum input limits of 35.00 mm on the AL, and the target refraction for the RBF 3.0 formula is restricted from − 2.50 D to 1.00 D, suggesting these may not have considered patients with high or extreme myopia who will require good near vision. Therefore, there is a critical need to develop an AI-based formula with significantly improved accuracy exclusively designed for highly myopic eyes.

The eXtreme Gradient Boosting (XGBoost) and support vector regression (SVR) are two popular and widely used machine learning models that provide applications in data classification and regression [15, 16]. The former derives from the gradient boosting decision tree and is recognized as highly efficient, portable and scalable [15]. The latter uses the principle of the support vector machine method, featured by its robustness and accuracy in data prediction [16]. Using these two models, we developed a novel IOL power calculation formula exclusively for refractive prediction in highly myopic eyes, and compared its performance with the BUII, EVO 2.0, Kane, Pearl-DGS and RBF 3.0 formulas.

## Methods

The study was approved by the Institutional Review Board of the Eye & Ear, Nose, and Throat (ENT) Hospital of Fudan University (ID: 2020005). Written informed consent was obtained from all participants for the use of their clinical data. All procedures adhered to the tenets of the Declaration of Helsinki.

### Subjects

High myopia was defined as AL ≥ 26.00 mm. The internal dataset was collected from the Eye & ENT Hospital of Fudan University from Jan 2019 to Aug 2021, and the external test datasets were collected from the Shanghai Aier Eye Hospital and the Eye Hospital of Wenzhou Medical University. Highly myopic eyes undergoing uneventful cataract surgeries with IOL implantation were reviewed. Eyes with complete preoperative biometric data and credible postoperative (one to two months after surgery) manifest refraction outcomes were included. Postoperative subjective refractive outcomes were assessed by a licensed optometrist and best corrected vision acuity was assessed at 5 m. Then, the refractions were standardized to a 6 m distance by adding − 0.03 D to the spherical equivalent. One eye was randomly selected if both eyes met the criteria. The exclusion criteria were eyes with postoperative best-corrected distance visual acuity (BCVA) less than 20/40, severe corneal opacity or other ocular diseases that may influence the accuracy of manifest refraction, and history of ocular trauma or surgery. In total, 1828 highly myopic eyes of 1828 patients were included in the internal dataset, and 151 highly myopic eyes of 151 patients were included in the external test dataset.

The complete preoperative biometric data included AL, flattest and steepest keratometry (K) values, anterior chamber depth (ACD, as measured from corneal epithelium to the lens), lens thickness (LT), and horizontal corneal diameter (CD), which were all measured by IOLMaster 700 (version 1.50, Carl Zeiss Meditec AG, Jena, Germany). In the internal dataset, the implanted IOL types included data obtained from the Tecnis ZCB00 (33 cases), Tecnis ZA9003 (29 cases), Zeiss CT ASPHINA 409MP (353 cases), Alcon SN60WF (34 cases), HumanOptics MC X11 ASP (658 cases), HumanOptics ASPIRA-aAY (39 cases), Rayner 920H (646 cases), and Ophtec B.V. 52501TW/TY (36 cases). In the external test dataset, the implanted IOL types included data from the Alcon SN6CWS (33 cases), Alcon SN60WF IQ (63 cases), Bausch & Lomb Akreos AO MI60 (28 cases), HumanOptics ASPIRA-aAY (7 cases), and Zeiss CT ASPHINA 509 M (20 cases). “A constants” were obtained from the IOL Con website (www.iolcon.org) [46] for each IOL type, as advised by Professors Hoffer and Savini [17].

### Dataset preparation

The internal dataset was randomly split into training dataset and internal test dataset with a fixed ratio (8:2, 1462 eyes in the training dataset and 366 eyes). The machine learning features used can be classified into three categories: (1) ocular biometrics: AL, flattest and steepest K values, ACD, LT, and CD; (2) IOL information: implanted IOL power and A constants suggested by the IOL Con website for each IOL type; (3) parameters after transformations: predicted refractions calculated by the Haigis and the SRK/T theoretical formulas [18, 19].

### Modeling

**Fig. 1:**
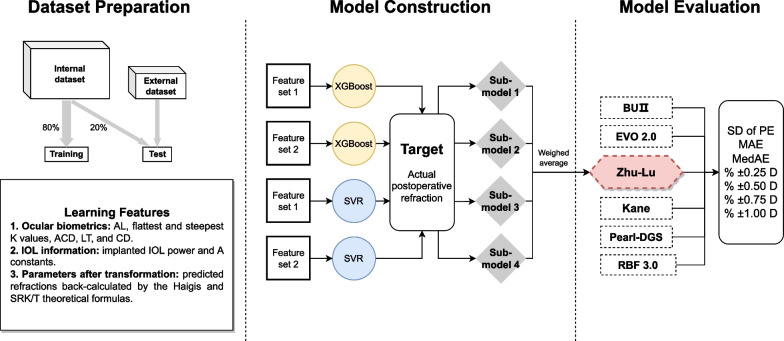
The flow diagram of model construction. In addition to all ocular biometric features and all IOL information features, feature set 1 incorporates results from the Haigis and SRK/T formulas, while feature set 2 only shows results from the Haigis formula. AL, axial length; CD, horizontal corneal diameter; LT, lens thickness; ACD, anterior chamber depth; IOL, intraocular lens; XGBoost, eXtreme Gradient Boosting; SVR, support vector regression; BUII, Barrett Universal II; EVO, Emmetropia Verifying Optical; RBF, Radial Basis Function; SD, standard deviation; PE, prediction error; D, diopter; MAE, mean absolute error; MedAE, median absolute error

The training dataset consisted of 1462 highly myopic eyes with complete information that was used for modeling. After a series of attempts at feature selection and combination, we constructed two sets of learning features. In addition to all ocular biometric features and all IOL information features, feature set 1 incorporates results from the Haigis and SRK/T formulas, while feature set 2 only contains results from the Haigis formula. Two supervised learning models, i.e., the XGBoost and the SVR, were trained with each of the two sets of learning features. The actual postoperative manifest refraction (presented as spherical equivalent [SE]) was set as the training target. Therefore, we obtained four sub-models, each of which can function independently for IOL power calculation in highly myopic eyes. To increase the model robustness, we adopted the weighted average of the calculation results from four sub-models and generated an assembled prediction model. Figure 1 shows the flow diagram of the model construction. Based on this assembled model, we built a novel IOL power calculation formula, named the Zhu-Lu formula (IOL power calculation formula for highly myopic eyes developed by Zhu and Lu). The software used in model construction was Python 3.7 with the scikit-learn package.

### Evaluation

The internal and external test datasets consisted of 366 and 151 highly myopic eyes, respectively. Due to the restriction of AL input (up to 35.00 mm) of the Kane and RBF 3.0 formulas, five cases in the internal test dataset and one case in the external test dataset were further excluded from analysis. The remaining 361 and 150 eyes in the internal and external test datasets were used. The prediction error (PE) was calculated as the actual postoperative refraction minus the predicted refraction back-calculated with the implanted IOL power using the BUII, EVO 2.0, Kane, Pearl-DGS, RBF 3.0 and Zhu-Lu formulas (URLs: additional sources [40–46]). The mean absolute errors (MAEs), median absolute errors (MedAEs) and percentages of eyes within ± 0.25 D, ± 0.50 D, ± 0.75 D, and ± 1.00 D of the PE were calculated and compared, as well as the cumulative percentages of eyes within different absolute errors. The formula performance index for each formula was calculated following recommendations by Hoffer et al. [17] which is based on four parameters: standard deviation (SD) of the PE, MedAE, the correlation between PE and AL (evaluated by the Pearson’s correlation test), and the inverse value of the percentage of eyes within ± 0.50 D of the PE. The higher the formula performance index, the more accurate the formula. Furthermore, accuracies were compared in subgroups according to different AL ranges (26.00–28.00 mm, 28.00–30.00 mm, and ≥ 30.00 mm).

### Applications

The website for the Zhu-Lu formula is currently available (URL: https://HM-ZLF.com/). Surgeons could easily calculate the optimal IOL power and predicted refraction by entering highly myopic eye’s ocular biometry data, target refraction, and IOL constants including A constant for the SRK/T formula and a0, a1, a2 constants for the Haigis formula.

### Statistics

Quantitative data were expressed as means ± SD and categorical data were displayed as proportions in demographics. After data normality was assessed with the Kolmogorov-Smirnov test, the one-way ANOVA test or the Kruskal-Wallis test was performed to analyze the normally or non-normally distributed continuous variables. Categorical data from demographics were compared using the χ^2^ test. The Friedman test with Bonferroni correction was used to assess the differences of MedAEs among formulas. The Cochran’s Q test with Bonferroni correction was conducted for comparisons of percentages of eyes within ± 0.25 D, ± 0.50 D, ± 0.75 D, and ± 1.00 D of the PE among formulas. The cumulative percentages of eyes within different absolute errors were compared using the log-rank test. Correlations between AL and PEs were assessed using the Pearson’s correlation analysis. A *P* value of less than 0.05 was considered statistically significant. All statistical analyses were performed using SPSS (version 26.0, IBM Corp., New York, US).

## Results

### Characteristics

The characteristics of the training dataset as well as the internal and external test datasets are shown in Table 1. There was no significant difference in eye laterality, gender, AL, AL subgroup distribution, flattest and steepest K values, ACD, LT, CD, implanted IOL power, and postoperative BCVA between the three datasets.

**Table 1:** Characteristics of highly myopic eyes in the training dataset, and the internal and external test datasets

Characteristics	Training dataset (n = 1462)	Internal test dataset (n = 361)	External test dataset (n = 150)	*P* value
Eye laterality (right/left)	756/706	186/175	78/72	0.736
Gender (male/female)	687/775	157/204	71/79	0.801
AL (mm)				0.517
Mean ± SD	29.18 ± 2.19	29.21 ± 2.06	29.01 ± 2.09	
Range	26.01–37.45	26.28–34.29	26.02–34.97	
AL subgroups				0.464
26.00–28.00 mm	536 (36.66%)	123 (34.07%)	54 (36.00%)	
28.00–30.00 mm	423 (28.93%)	118 (32.69%)	51 (34.00%)	
≥ 30.00 mm	503 (34.40%)	120 (33.24%)	45 (30.00%)	
Flattest K value (D)	43.30 ± 1.51	43.24 ± 1.56	43.55 ± 1.55	0.107
Steepest K value (D)	44.32 ± 1.61	44.29 ± 1.60	44.58 ± 1.61	0.141
ACD (mm)	3.42 ± 0.34	3.43 ± 0.34	3.42 ± 0.37	0.93
LT (mm)	4.44 ± 0.39	4.44 ± 0.40	4.38 ± 0.43	0.201
CD (mm)	11.75 ± 0.42	11.77 ± 0.46	11.77 ± 0.40	0.609
IOL power (D)	9.28 ± 5.05	9.31 ± 4.87	9.31 ± 4.41	0.941
Postop-BCVA (logMAR)	0.05 ± 0.03	0.04 ± 0.03	0.03 ± 0.04	0.837

*AL* = axial length; *SD* = standard deviation; *D* = diopter; *ACD* = anterior chamber depth; *LT* = lens thickness; *CD* = horizontal corneal diameter; *IOL* = intraocular lens; *Postop-BCVA* = postoperative best-corrected visual acuity; *logMAR* = logarithm of the minimal angle of resolution

### Accuracy evaluation

Table 2 demonstrates the prediction accuracy of the six IOL calculation formulas in the internal and external test datasets. In the internal test dataset, the SD values of the six IOL calculation formulas, in order of lowest to highest, were Zhu-Lu (0.46 D), RBF 3.0 (0.51 D), BUII (0.54 D), Kane (0.55 D), EVO 2.0 (0.56 D) and Pearl-DGS (0.71 D). The Zhu-Lu formula showed significantly lower MedAEs than BUII, EVO 2.0, Kane and Pearl-DGS formulas (Friedman test with Bonferroni post hoc analysis, all *P* < 0.05). The IOL formula performance indexes, in order of highest to lowest, were Zhu-Lu, RBF 3.0, Kane, EVO 2.0, BUII and Pearl-DGS formulas. In the external test dataset, the SD values of the six IOL calculation formulas, in order of lowest to highest, were Zhu-Lu (0.50 D), Kane (0.52 D), EVO 2.0 (0.53 D), Pearl-DGS (0.53 D), BUII (0.57 D) and RBF 3.0 (0.57 D). The Zhu-Lu formula showed significantly lower MedAEs than Kane, Pearl-DGS and RBF 3.0 formulas (Friedman test with Bonferroni post hoc analysis, all *P* < 0.05). As for IOL Formula Performance Indices, the Zhu-Lu formula performed similar to the BUII and EVO 2.0, but better than the RBF 3.0, Kane and Pearl-DGS formulas.

**Table 2:** Prediction outcomes of the BUII, EVO 2.0, Kane, Pearl-DGS, RBF 3.0 and Zhu-Lu formulas in highly myopic eyes

Parameters	BUII	EVO 2.0	Kane	Pearl-DGS	RBF 3.0	Zhu-Lu	*P* value
*Internal test dataset (n = 361)*							
PE (D)							
Mean	− 0.11	− 0.14	− 0.31	0.14	− 0.10	0.005	
SD	0.54	0.56	0.55	0.71	0.51	0.46	
MAE ± SD (D)	0.46 ± 0.30	0.46 ± 0.34	0.49 ± 0.39	0.55 ± 0.47	0.38 ± 0.35	0.34 ± 0.31	
MedAE (D)	0.43*	0.40*	0.40*	0.45*	0.29	0.26	< 0.001
IOL formula performance index	0.048	0.048	0.056	0.040	0.065	0.071	
*External test dataset (n = 150)*							
PE (D)							
Mean	− 0.01	− 0.02	− 0.21	0.20	− 0.06	0.05	
SD	0.57	0.53	0.52	0.53	0.57	0.50	
MAE ± SD (D)	0.43 ± 0.38	0.40 ± 0.35	0.45 ± 0.34	0.45 ± 0.34	0.44 ± 0.37	0.38 ± 0.34	
MedAE (D)	0.34	0.32	0.37*	0.40*	0.36*	0.30	0.001
IOL formula performance index	0.063	0.063	0.052	0.050	0.056	0.063	

*BUII* = Barrett Universal II; *EVO* = Emmetropia Verifying Optical; *RBF* = Radial Basis Function; *PE* = prediction error; *SD* = standard deviation; *D* = diopters; *MAE* = mean absolute error; *MedAE* = median absolute error; *IOL* = intraocular lens

^*^P < 0.05 when compared with the Zhu-Lu formula using the Friedman test with Bonferroni post hoc analysis

**Fig. 2:**
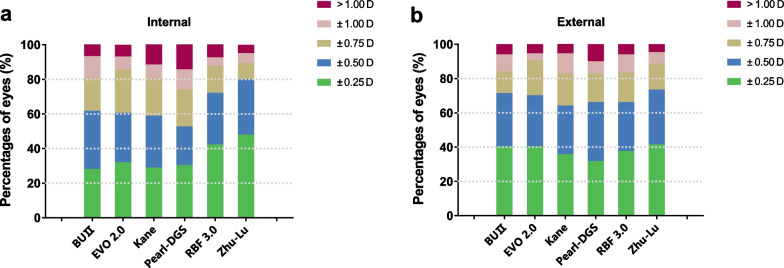
Percentages of eyes within ± 0.25 D, ± 0.50 D, ± 0.75 D, and ± 1.00 D of the prediction errors of each formula in the internal (**a**) and external (**b**) test datasets. BUII, Barrett Universal II; EVO, Emmetropia Verifying Optical; RBF, Radial Basis Function; D, diopter

**Fig. 3:**
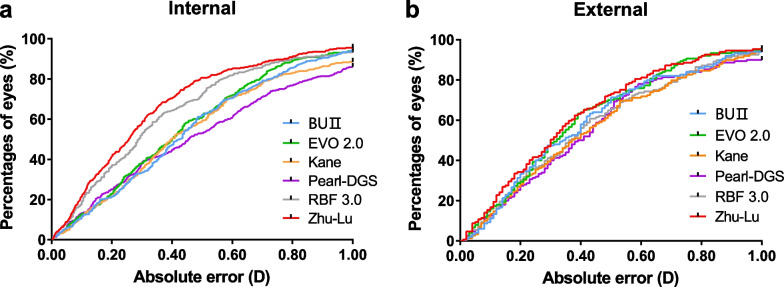
Cumulative distribution function curves of absolute errors of each formula in highly myopic eyes in the internal (**a**) and external (**b**) test datasets. BUII, Barrett Universal II; EVO, Emmetropia Verifying Optical; RBF, Radial Basis Function; D, diopter

The distribution of the PE in both internal and external test datasets using histograms are shown in Fig. 2. In the internal test dataset, the Zhu-Lu formula showed the highest percentages of eyes within ± 0.50 D of the PE (80.61%), followed by RBF 3.0 (72.85%), BUII (62.33%), EVO 2.0 (61.22%), Kane (59.56%) and Pearl-DGS (53.19%). The BUII, EVO 2.0, Zhu-Lu and RBF 3.0 formulas had comparable percentages of eyes within ± 1.00 D of the PE, higher than the Kane and Pearl-DGS formulas (all *P* < 0.05). In the external test dataset, the Zhu-Lu formula also showed the highest percentages of eyes within ± 0.50 D of the PE (72.85%), followed by BUII (70.86%), EVO 2.0 (69.54%), Pearl-DGS (65.56%), RBF 3.0 (65.56%) and Kane (63.58%). All formulas predicted over 90% of eyes with PE within ± 1.00 D. Figure 3 shows cumulative distribution function curves of six formulas in both test datasets. In the internal test dataset, cumulative distribution function curves show that the distribution patterns of the absolute errors were different among formulas and the Zhu-Lu formula performed better than the other formulas (log-rank test, all *P* < 0.05) except for the RBF 3.0 formula (*P* = 0.058). In the external test data, the cumulative distribution function curve of the Zhu-Lu formula was comparable to the BUII, EVO 2.0 and RBF 3.0 formulas (logrank test, *P* = 0.219, 0.548 and 0.112, respectively), while significantly better than the Kane and Pearl-DGS formulas (both *P* < 0.05).

### Subgroup analysis

The accuracies of the six formulas were further compared in different AL subgroups (Table 3). In the internal test dataset, the Zhu-Lu formula had significantly lower MedAEs than the BUII, EVO 2.0, Kane and Pearl-DGS formulas in all three AL subgroups (Friedman test with Bonferroni post hoc analysis, all *P* < 0.05). When compared to the RBF 3.0 formula, no significant difference was found. In the external test dataset, the Zhu-Lu formula had a significantly lower MedAE than the BUII, EVO 2.0 and Pearl-DGS formulas in the 26.00–28.00 mm AL subgroup (all *P* < 0.05), and statistically comparable with the Kane and RBF 3.0 formulas. In the 28.00–30.00 mm AL subgroup, there were no significant differences among formulas. In the AL ≥ 30.00 mm subgroup, the Zhu-Lu formula had a significantly lower MedAE than the Kane, RBF 3.0 and Pearl-DGS formulas (all *P* < 0.05).

**Table 3:** Comparison of the prediction errors using the BUII, EVO 2.0, Kane, Pearl-DGS, RBF 3.0 and Zhu-Lu formulas in different axial length subgroups of highly myopic eyes

Parameters	BUII	EVO 2.0	Kane	Pearl-DGS	RBF 3.0	Zhu-Lu	*P* value
*Internal test dataset (n = 361)*							
26.00–28.00 mm							
Number	123						
MAE ± SD (D)	0.42 ± 0.29	0.46 ± 0.38	0.48 ± 0.41	0.42 ± 0.39	0.39 ± 0.38	0.35 ± 0.34	-
MedAE (D)	0.39*	0.40*	0.37*	0.30*	0.29	0.26	< 0.001
28.00–30.00 mm							
Number	118						
MAE ± SD (D)	0.47 ± 0.31	0.47 ± 0.34	0.51 ± 0.38	0.49 ± 0.37	0.39 ± 0.35	0.32 ± 0.30	-
MedAE (D)	0.44*	0.42*	0.44*	0.42*	0.30	0.23	< 0.001
≥ 30.00 mm							
Number	120						
MAE ± SD (D)	0.48 ± 0.29	0.43 ± 0.32	0.50 ± 0.38	0.76 ± 0.56	0.35 ± 0.30	0.34 ± 0.31	-
MedAE (D)	0.44*	0.39*	0.40*	0.66*	0.29	0.27	< 0.001
*External test dataset (n = 150)*							
26.00–28.00 mm							
Number	54						
MAE (D) ± SD	0.47 ± 0.46	0.47 ± 0.44	0.45 ± 0.39	0.42 ± 0.28	0.44 ± 0.43	0.37 ± 0.38	-
MedAE (D)	0.42*	0.35*	0.32	0.40*	0.31	0.29	0.036
28.00–30.00 mm							
Number	51						
MAE ± SD (D)	0.40 ± 0.29	0.35 ± 0.26	0.40 ± 0.29	0.40 ± 0.30	0.39 ± 0.28	0.37 ± 0.32	-
MedAE (D)	0.35	0.29	0.41	0.33	0.37	0.30	0.373
≥ 30.00 mm							
Number	45						
MAE ± SD (D)	0.40 ± 0.35	0.37 ± 0.30	0.50 ± 0.32	0.55 ± 0.43	0.50 ± 0.38	0.39 ± 0.31	-
MedAE (D)	0.28	0.30	0.40*	0.46*	0.42*	0.33	0.002

*BUII* = Barrett Universal II; *EVO* = Emmetropia Verifying Optical; *RBF* = Radial Basis Function; *SD* = standard deviation; *D* = diopters; *MAE* = mean absolute error; *MedAE* = median absolute error; *IOL* = intraocular lens

^*^P < 0.05 when compared to the Zhu-Lu formula using the Friedman test with Bonferroni post hoc analysis

**Fig. 4:**
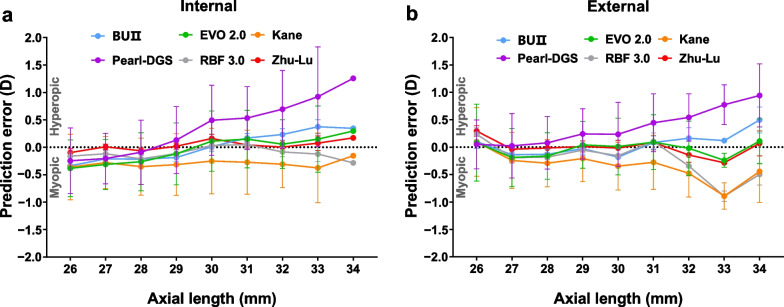
Prediction errors (mean ± standard deviation) versus axial length of each formula in highly myopic eyes in the internal (**a**) and external (**b**) test datasets. BUII, Barrett Universal II; EVO, Emmetropia Verifying Optical; RBF, Radial Basis Function; D, diopter

Figure 4 demonstrates the effect of AL on PEs in both internal and external test datasets. The PEs of the Zhu-Lu formula were close to zero especially in extremely myopic eyes.

In addition, we further evaluated the performance of the Zhu-Lu formula in eyes with AL > 35.00 mm, which have been excluded from the internal and external test datasets due to the restrictions of RBF 3.0 and Kane formulas (Additional file 1: Table S1). It revealed that the MAE was 0.27 ± 0.25 D, and the MedAE achieved 0.23 D. Furthermore, 50.00%, 83.33%, 83.33% and 100.00% of them were within ± 0.25 D, ± 0.50 D, ± 0.75 D and ± 1.00 D of the PE, respectively.

## Discussion

Current IOL power calculation formulas are not accurate enough for highly myopic eyes, especially in light of the increasing demand for multifocal IOL implantations [20, 21]. The newer generation of AI-based formulas, though achieving improved accuracies, still have some limitations when applied to highly myopic eyes [2, 22]. In this study, using the XGBoost and SVR machine learning models, we developed the novel Zhu-Lu formula, which demonstrated significantly improved accuracy compared to other AI-based formulas in highly myopic eyes.

The dataset for developing the Zhu-Lu formula is derived from the largest cohort (1462 eyes in the training dataset with AL ranging from 26.01 to 37.45 mm) of highly myopic cataract patients. For a machine learning based formula, plenty and credible training data are crucial to its accuracy and stability. Furthermore, the XGBoost and SVR algorithms are both highly accurate in data prediction and have helped with numerous situations in ophthalmology, such as diabetic retinopathy diagnosis, implantable collamer lens size selection, and post-cataract-surgery focus depth prediction [23–25]. Using the XGBoost, our group has previously developed an enhanced calculator for the BUII formula, which demonstrated improved prediction accuracy in highly myopic eyes [26, 27]. Yet this enhanced calculator might be influenced by the internal optimizations of the BUII formula itself, which is still unpublished to the public. Conversely, the current Zhu-Lu formula is based on theoretical formulas of SRK/T and Haigis and other feature transformations carried out inside the model, which can be used independently in clinical practice. Also, except for the XGBoost, the Zhu-Lu formula also adopted the SVR model [28], which may explain the improved performance compared to formulas using single algorithms such as the RBF 3.0. Notably, in this study, an assembled model using both algorithms and weighted average strategy of four sub-models was constructed, which could further enhance the robustness of Zhu-Lu formula.

A number of AI-based IOL calculation formulas have been developed in recent years. The BUII formula, an updated version of BU formula, was introduced in 2010 by Graham D Barrett and showed promising results in myopic eyes [10, 29, 30]. The EVO 2.0 formula generates an emmetropia factor for each eye and its 2.0 version is considered to have better prediction in long eyes [31–33]. The Kane formula was developed on the basis of both regression and AI elements and yielded decent outcomes in previous studies [33–35]. The Pearl-DGS formula, published in 2019, was an AI-based formula developed utilizing the support vector machine and multilayer neural networks and showed promising accuracy according to a recent study [36]. The RBF 3.0 formula was a data-driven IOL power calculator that had been continuously expanding its database. The 3.0 version was recently released, with reported better performance than the 2.0 version [7].

When applied to highly myopic eyes, the reported MedAEs of these formulas ranged from 0.22 to 0.33 D [22, 31, 34, 35, 37, 38]. In this study, we found that the Zhu-Lu formula outperformed the BUII, EVO 2.0, Kane and Pearl-DGS formulas in the internal test dataset. In the external test dataset, the Zhu-Lu formula had a MedAE that was significantly lower than the Pearl-DGS and RBF 3.0 formulas. Notably, the Zhu-Lu formula had a significantly lower MedAE than the BUII and EVO 2.0 formulas in the 26.00 to 28.00 mm subgroup. However, in the ≥ 30.00 mm subgroup, the Zhu-Lu formula performed similar to the BUII and EVO 2.0 formulas but outperformed the Kane and RBF 3.0 formulas. This phenomenon highlights the advantage of prediction stability of the Zhu-Lu formula in different AL ranges. Furthermore, the PEs of the Zhu-Lu formula were around zero and remained stable as AL elongates (Fig. 4).

The other noteworthy point of the Zhu-Lu formula is the improved percentages of eyes within ± 0.25 D and ± 0.50 D of the PE among highly myopic eyes, as compared to other formulas. For multifocal IOL implantations, pursuing higher percentage of eyes within ± 0.25 D or ± 0.50 D is important for satisfied postoperative distant and near vision [39]. Therefore, highly myopic eyes requiring multifocal IOL implantations may benefit a lot from the novel Zhu-Lu formula with better predictability and postoperative refractive outcomes.

Additionally, the performance of the Zhu-Lu formula in eyes with AL > 35 mm also remained stably promising. As no restrictions for target refraction or AL input limitations were set for the Zhu-Lu formula, the Zhu-Lu formula can apply to a wider range of highly myopic patients, especially those seeking better near visions. Overall, we suggest that the Zhu-Lu formula be used in highly or especially extremely myopic eyes.

## Conclusions

In conclusion, we developed a novel Zhu-Lu formula exclusively for IOL power calculation and refractive prediction in highly myopic eyes. The model was derived from an extensive database, incorporated various biometric features, and took advantage of the XGBoost and the SVR machine learning algorithms. In both internal and external test datasets, the Zhu-Lu formula presented more promising outcomes in highly myopic eyes over the other AI-based formulas.

## Supplementary Information

**Additional file 1: Table S1.** Performance of the Zhu-Lu formula in eyes with AL > 35 mm.

## Data Availability

The data that support the findings of this study are available from the corresponding author upon reasonable request.

## Abbreviations

ACD: Anterior chamber depth
AI: Artificial intelligence
AL: Axial length
BCVA: Best-corrected distance visual acuity
BUII: Barrett Universal II
CD: Horizontal corneal diameter
D: Diopter
EVO: Emmetropia verifying optical
IOL: Intraocular lens
K: Keratometry
LT: Lens thickness
MAE: Mean absolute error
MedAE: Median absolute error
PE: Prediction error
RBF: Radial Basis Function
SD: Standard deviation
SE: Spherical equivalent
SVR: Support vector regression
XGBoost: EXtreme Gradient Boosting
