# Supplementary material for: The Zhu-Lu formula: a machine learning-based intraocular lens power calculation formula for highly myopic eyes
Source: Eye Vis (Lond). 2023 Jun 1;10:26. doi: 10.1186/s40662-023-00342-5 (PMC10233923; doi:10.1186/s40662-023-00342-5)
Supplement: Supplementary file 1 — Additional file 1: Table S1. Performance of the Zhu-Lu formula in eyes with AL > 35 mm. [file 40662_2023_342_MOESM1_ESM.docx]

**Table S1.** Performance of the Zhu-Lu formula in eyes with AL > 35 mm.

| Parameters | Zhu-Lu formula |
| --- | --- |
| Number of eyes | 6 |
| AL (mm) |  |
| Mean ± SD | 35.73 ± 0.54 |
| Range | 35.17 – 36.42 |
| MAE ± SD (D) | 0.27 ± 0.25 |
| MedAE (D) | 0.23 |
| Percentage of eyes within range of PE |  |
| ±0.25 D | 50.00% (3/6) |
| ±0.50 D | 83.33% (5/6) |
| ±0.75 D | 83.33% (5/6) |
| ±1.00 D | 100.00% (6/6) |

AL = axial length; SD = standard deviation; D = diopter; MAE = mean absolute error; MedAE = median absolute error; PE = prediction error.
